# Diagnostic et prévalence du syndrome métabolique chez les diabétiques suivis dans un contexte de ressources limitées: cas du Burkina-Faso

**DOI:** 10.11604/pamj.2014.19.364.3741

**Published:** 2014-12-09

**Authors:** Yaméogo Téné Marceline, Sombié Issiaka, Kyélem Carole Gilberte, Rouamba Nadège, Ouédraogo Sampawindé Macaire, Yaméogo Aimé Arsène, Lankoandé Djingri, Sawadogo Apollinaire, Drabo Youssouf Joseph

**Affiliations:** 1 Institut supérieur des Sciences de la Santé (INSSA), Bobo-Dioulasso, Burkina-Faso; 2 CHU Sourô Sanou de Bobo Dioulasso, Burkina-Faso; 3 Organisation ouest-africaine de la Santé, Burkina-Faso; 4 Unité de Formation et de Recherche en Sciences de la santé, Ouagadougou, Burkina-Faso; 5 CHU Yalgado Ouédraogo de Ouagadougou, Burkina-Faso

**Keywords:** Syndrome métabolique, Burkina-Faso, diabétiques, metabolic syndrome, Burkina-Faso, diabetics

## Abstract

**Introduction:**

Les conséquences du syndrome métabolique impliquent son diagnostic effectif pour une prise en charge globale des comorbidités dépistées. Objectif: Déterminer la capacité à diagnostiquer le syndrome métabolique en routine, sa prévalence chez les diabétiques, leurs connaissances et pratiques vis-à-vis du risque cardio-métabolique.

**Méthodes:**

Il s'est agi d'une étude transversale auprès de 388 diabétiques au CHU de Bobo-Dioulasso. Les critères de la fédération internationale du diabète (2009) ont été utilisés.

**Résultats:**

l’âge moyen était de 53,5±13,5 ans, le sex ratio de 0,7. L'obésité abdominale était présente dans 61,9% des cas; L'HTA l’était dans 56,4% des cas. La prescription du bilan lipidique a été documentée dans 55,4% des cas pour le HDL et 56,2% pour les triglycérides pour un taux de réalisation de 49,3% et 62,9%. Le taux de dépistage des critères lipidiques était de 26,8%. Un taux de HDL bas a été noté dans 46 cas (43,4%) et une hypertriglycéridémie dans 24 cas (17,6%). In fine, la prévalence du syndrome métabolique était de 48,9% (n = 190). Seuls 27,4% savaient que d'autres facteurs de risque cardiovasculaire pouvaient être associés au diabète et seulement 6,7% pratiquaient une activité physique régulière.

**Conclusion:**

Malgré la faible contribution du laboratoire, le syndrome métabolique est fréquent parmi nos diabétiques. Les patients sont peu sensibilisés sur le risque vasculaire et la pratique d'une activité physique régulière reste faible. Un programme d’éducation adaptée contribuerait à un meilleur dépistage et à une prise en charge optimale des cas.

## Introduction

Le diabète est une maladie métabolique à l'origine de complications micro et macrovasculaires qui font toute sa gravité [1, 2]. L'association d'autres facteurs augmente le risque vasculaire chez le diabétique [3]. Au nombre de ces facteurs, il y a le syndrome métabolique qui précède ou accompagne le diabète. Le syndrome métabolique associe des troubles souvent modérés, d'origine glucidique, lipidique et vasculaire et une surcharge pondérale, qui agissent en synergie pour provoquer un diabète de type 2; ils prédisposent à l'athérosclérose et à ses événements cliniques [4, 5]. En dépit de l'existence de différents critères de définition [6, 7], les études s'accordent sur l'importance de sa prévalence au sein de la population en général et des diabétiques en particulier [8–10]. Ses conséquences impliquent la nécessité d'un diagnostic effectif des cas afin d'assurer une prise en charge intégrée de tous les facteurs de risque cardiovasculaire dépistés. En l'absence de données sur l'ampleur du phénomène parmi les diabétiques au Burkina Faso, nous avons réalisé cette étude afin de déterminer dans les conditions de travail de routine, l'effectivité du dépistage des critères biologiques (lipidiques), la prévalence du syndrome métabolique ainsi que les connaissances et les pratiques des diabétiques vis-à-vis du risque cardio-métabolique.

## Méthodes

Nous avons mené une étude transversale descriptive sur un échantillon de 388 diabétiques consentants, et recrutés de façon continue. L'enquête a concerné les diabétiques ayant au moins un an d'ancienneté du diabète et suivis au CHU de Bobo-Dioulasso. Les critères de la fédération internationale du diabète (FID) 2009 ont été utilisés pour la définition du syndrome métabolique [7], c'est-à-dire la présence de 3 au moins des 5 critères suivants: - une obésité abdominale: correspondant à un tour de taille supérieur ou égal à 94 cm chez l'homme et 80 cm chez la femme; - une triglycéridémie supérieure ou égale à 1,50 g/l (ou 1,7mmol/l) et/ou la prise d'un traitement hypolipémiant spécifique; - un taux de HDL-cholestérol inférieur ou égal à 0,40g/l (1,03 mmol/l) chez l'homme et 0,50 g/l (1,29 mmol/l) chez la femme et/ou la prise d'un traitement hypolipémiant spécifique; - une HTA, définie par une pression artérielle supérieure ou égale à 130 / 85 mm Hg et/ou la prise d'un traitement antihypertenseur; - une glycémie à jeun élevée, supérieure ou égale à 1 g/l (5,6 mmol/l) ou traitement antidiabétique. Étant entendu que tous nos patients étaient diabétiques, le diagnostic du syndrome métabolique a été porté en présence de deux au moins des quatre autres critères (obésité abdominale, HTA, triglycérides élevées, cholestérol-HDL bas). Une fiche individuelle a permis la collecte des données cliniques et paracliniques. Un questionnaire, portant sur la connaissance des autres facteurs de risque cardiovasculaire et la pratique d'une activité physique régulière, a également été inclus dans la fiche et administré à chaque diabétique par interview. L'analyse a consisté en la production de statistiques descriptives. Le test du chi2 a été utilisé au seuil de signification de 5%.

## Résultats

### Caractéristiques sociodémographiques

L’âge moyen des 388 patients était de 53,5±13,5 ans. Les diabétiques de 40 ans et plus représentaient 86,1% des cas. Les femmes au nombre de 225, représentaient 58,0% de l'effectif, soit un sex ratio de 0,7. Sur le plan de la profession, on distinguait, 153 femmes au foyer (39,4%), 69 travailleurs salariés (17,8%), 47 commerçants (12,1%), 46 cultivateurs/bergers (11,8%), 46 retraités (11,8%), 6 élèves/étudiants (1,8%) et 20 sans-emploi (5,2%). Seuls 6 diabétiques (1,5%) bénéficiaient d'une assurance-maladie. Les diabétiques scolarisés représentaient 43,6% des cas (n = 169) et ceux résidant en milieu urbain 85,6% (n = 332).

### Les critères cliniques

Deux cent cinquante-deux diabétiques (64,9%) avaient une obésité abdominale. Cette obésité touchait plus souvent les femmes (88,4%) que les hommes (32,5%), p<0,01. On notait que 219 diabétiques (56,4%) présentaient une HTA. La proportion d’hypertendus chez les femmes (60%) était plus importante que chez les hommes (51,5%) sans différence statistiquement significative (p=0,09).

### Les critères paracliniques lipidiques

Le tableau 1 montre la prescription et la réalisation des examens de dépistage des anomalies lipidiques une fois au moins sur les 12 derniers mois. On note que les taux de prescriptions et de réalisations de ces examens variaient entre 50 et 60% Le taux de dépistage de la dyslipidémie était d’environ 27% pour le HDL et 35% pour les triglycérides tant chez les diabétiques hypertendus que chez ceux ayant une obésité abdominale (Tableau 1). Au total, sur les 388 diabétiques, les critères lipidiques (c'est-à-dire la réalisation des deux examens: HDL-cholestérol + triglycérides) ont été évalués chez seulement 104 cas soit 26,8%. Le résultat de l'exploration lipidique a montré un taux de HDL-cholestérol bas dans 46 cas (43,4%) et une hypertriglycéridémie dans 24 cas (17,6%).

**Tableau 1:** Dépistage des critères lipidiques chez les diabétiques suivis au CHU de Bobo – Dioulasso

Item	Cholestérol	HDL	Triglycérides	
	Nombre	Taux (%)	Nombre	Taux (%)
Prescription générale (N = 388)	215	55,4	218	56,2
Réalisation de l'examen prescrit	106	49,3	136	62,4
Dépistage général (N = 388)	106	27,3	136	35,1
Dépistage chez les diabétiques hypertendus (n = 219)	61	27,9	77	35,2
Dépistage chez les diabétiques avec obésité abdominale (n = 252)	69	27,4	86	34,1

### Prévalence du syndrome métabolique

Selon les critères de la FID, 190 patients associaient au diabète au moins 2 autres critères et avaient ainsi un syndrome métabolique, soit une prévalence de 48,9%. Parmi eux, 32 (16,8%) avaient un syndrome métabolique sévère: 30 (15,8%) cumulaient 4 critères et 2 (1,1%) tous les 5 critères. Cette prévalence peut être considérée comme minimale puisque 89 diabétiques (22,9% de l'effectif total) ayant un 2ème critère (32 hypertendus et 57 avec une obésité abdominale) n'avaient pas eu de dépistage de la dyslipidémie. Le tableau 2 donne la répartition du syndrome métabolique selon les caractéristiques socioéconomiques, les connaissances et la pratique du sport chez les diabétiques. On notait que la prévalence du syndrome métabolique était significativement plus importante chez les diabétiques âgés de 40 ans et plus par rapport aux diabétiques de moins de 30 ans, chez les femmes par rapport aux hommes et chez les diabétiques citadins par rapport aux ruraux. On ne notait pas de différence de prévalence selon la scolarisation, la connaissance des autres facteurs de risque et la pratique d'une activité physique (Tableau 2).

**Tableau 2:** Prévalence du syndrome métabolique selon les caractéristiques des diabétiques suivis au CHU de Bobo – Dioulasso

N°	Caractéristiques des diabétiques		Prévalence du syndrome métabolique en%		
1	Age	moins de 30 ans	13,0	(3/23)	< 0,01
		30 ans et plus	51,0	(186/365)	
2	Sexe	Masculin	33,7	(55/163)	< 0,01
		Féminin	60,0	(135/225)	
3	Scolarisés	Oui	53,3	(90/169)	0,13
		Non	45,7	(100/219)	
4	Résidence	Urbaine	53,3	(177/332)	< 0,01
		Rurale	23,2	(13/56)	
5	Activité physique	Oui	46,2	(12/26)	0,76
		Non	49,2	(178/362)	
6	Connaissance des autres FRCV	Oui	55,8	(58/104)	0,10
		Non	46,8	(132/284)	

### Connaissances et pratiques des diabétiques vis-à-vis du risque cardio-métabolique

Seuls 27,4% (n = 104) des diabétiques savaient que d'autres facteurs de risque cardiovasculaire tel l’âge, la sédentarité, l'HTA, la dyslipidémie, l'obésité pouvaient être associé au diabète. Vingt-six patients (6,7% des cas) ont déclaré pratiquer une activité physique régulière autre que de routine. Il s'agissait du footing dans la majorité des cas (n = 24). Elle était pratiquée seule ou associée à la gymnastique et/ou au vélo. Dans les 2 autres cas, il s'agissait de course à pied et de natation. La durée hebdomadaire moyenne de l'activité physique était de 01 heure 54mn ± 48mn, avec des extrêmes de 30 mn et 04 heures.

## Discussion

Il est ressorti de notre travail que la prescription et la réalisation des examens biologiques entrant dans l’évaluation du syndrome métabolique n’étaient pas systématiques chez tous les diabétiques dans notre pratique. Malgré cette faiblesse, le syndrome métabolique a été dépisté chez 48,9% des diabétiques avec une prédominance chez les sujets de 30 ans et plus, les femmes et les résidents en milieu urbain. Enfin seulement 27,4% (n = 104) des diabétiques savaient que d'autres facteurs de risque cardiovasculaire tel l’âge, la sédentarité, l'HTA, la dyslipidémie et l'obésité pouvaient être associés au diabète. Plusieurs facteurs pourraient expliquer la non évaluation systématique du syndrome métabolique dans notre pratique. Le premier facteur est lié à l'archivage des bulletins des examens chez les diabétiques dont la plupart sont non alphabétisés. Le deuxième facteur serait lié au coût des examens de suivi pour une population dont 40% sont pauvres et dans un contexte de faible existence d'aide aux diabétiques (Yaméogo et al, article soumis). Dans ce contexte, la réalisation du bilan lipidique annuel pourrait être considérée comme secondaire, les médicaments antidiabétiques et le contrôle glycémique étant privilégiés [11–13] Pourtant, la dyslipidémie est d'observation fréquente dans la population diabétique de type 2. C'est un facteur de risque majeur de survenue d’évènements cardiovasculaires; sa correction, par les statines notamment, permet de réduire la morbi-mortalité cardiovasculaire chez le diabétique de type 2, jusqu’à 37% comparativement au placebo, comme observé dans l’étude CARDS (Collaborative Atorvastatin Diabetes Study) [4, 14, 15]. Les diabétiques devraient être sensibilisés à ce risque pour une meilleure adhésion à la prescription du bilan lipidique. Il faut cependant souligner que les prescripteurs devraient l’être davantage pour que l'exploration lipidique soit systématique pour tous les diabétiques. En effet, le taux de réalisation du bilan lipidique, de 49% pour le HDL à 62% pour la triglycéridémie pouvait être considéré comme satisfaisant. Dans un contexte plus nanti, l’étude ENTRED en France rapportait en effet un taux de réalisation de 76% pour le dosage annuel des lipides [16].

Cette étude a montré que le syndrome métabolique touchait au moins 48,9% des diabétiques suivis au CHU de Bobo-Dioulasso. Notre résultat s'inscrit dans les tendances observées dans la littérature et pourrait même s'avérer plus important vu que 89 diabétiques, présentant une HTA ou une obésité abdominale, n'avaient pas de bilan lipidique documenté et n'ont donc pas été pris en compte. En effet, dépendamment des études et des critères de définition utilisés, la prévalence du syndrome métabolique varie d'au moins 34 à 80% au sein des diabétiques, traduisant ainsi l'importance du problème [10, 17, 18]. L'obésité abdominale serait le déterminant de cette forte prévalence [19]. Dans notre étude, 64% des diabétiques en souffraient et plus particulièrement les femmes (88,4% des cas). On observait en effet une prédominance du syndrome métabolique chez les femmes, ainsi que rapporté également par d'autres études [17]. La problématique du syndrome métabolique est surtout celle de sa prise en charge globale, le risque cardiovasculaire étant avéré. En effet, l'hyperglycémie n'est pas seule en cause dans l'augmentation du risque cardiovasculaire chez le diabétique: aussi bien l'HTA que la dyslipidémie et l'insulinorésistance sont impliquées [20]. Cela explique la nécessité de dépister les différentes composantes de ce syndrome afin d'assurer une prise en charge globale des comorbidités diagnostiquées, notamment les facteurs de risque modifiables [20]. Cette prise en charge, outre les différents traitements médicamenteux (antidiabétiques, hypolipémiants, antihypertenseurs), devraient inclure un régime alimentaire adapté et une activité physique régulière. Zeber et al ont en effet montré qu'une attention particulière apportée au régime et aux médicaments, réduisait de 39 à 44% le risque de survenue d’évènements cardiovasculaires [21]

Dans notre étude, seulement 6% des diabétiques ayant un syndrome métabolique pratiquaient une activité physique régulière autre que de routine. Cette pratique semble en effet peu fréquente dans notre contexte, surtout en milieu urbain. Une étude réalisée au Cameroun montrait en effet que résider en milieu urbain était corrélé à une plus faible dépense énergétique et à une plus forte prévalence du syndrome métabolique par rapport à la résidence rurale [22]. Il est toutefois nécessaire d’éduquer les diabétiques pour qu'ils s'adonnent à plus de sport (ou activité physique régulière). Une meilleure connaissance de la maladie pourrait en effet être déterminante pour l'auto-mise en œuvre des bonnes pratiques hygiéno-diététiques. Il est prouvé en effet qu'un bon niveau de connaissance du diabète est corrélé aux bonnes pratiques de prise en charge [23, 24]. Seulement 27% de nos diabétiques connaissaient les facteurs associés au risque cardio-métabolique. Il n'existe pas actuellement une unité polyvalente de prise en charge du diabète au CHUSS. La mise en œuvre d'un programme d’éducation avec une équipe d'agents dédiée et formée contribuerait à améliorer le niveau de connaissance des patients vis-à-vis du diabète et des autres FRCV.

## Conclusion

Malgré la faible contribution du laboratoire dans son diagnostic, le syndrome métabolique est fréquent parmi les diabétiques suivis au CHUSS, et touche particulièrement les femmes. Ces patients sont toutefois peu sensibilisés sur le risque vasculaire et ont une faible pratique d'une activité physique régulière. Un programme d’éducation adaptée contribuerait à un meilleur dépistage et à une prise en charge optimale des cas.
